# Sex-dependent neuronal effects of α-synuclein reveal that GABAergic transmission is neuroprotective of sleep-controlling neurons

**DOI:** 10.1186/s13578-023-01105-4

**Published:** 2023-09-14

**Authors:** Altair Brito Dos Santos, Siganya Thaneshwaran, Lara Kamal Ali, César Ramón Romero Leguizamón, Yang Wang, Morten Pilgaard Kristensen, Annette E. Langkilde, Kristi A. Kohlmeier

**Affiliations:** 1 Department of Drug Design and Pharmacology, Faculty of Health and Medical Sciences, University of Copenhagen, Copenhagen, 2100 Denmark; 2 Dept of Neuroscience, University of Copenhagen, Copenhagen, 2200 Denmark; 3 Academic Services, Vaerløse, 3500 Denmark

**Keywords:** Sleep disorders, Cholinergic, Laterodorsal Tegmentum, α -synucleinopathies, neurodegenerative disease

## Abstract

**Background:**

Sleep disorders (SDs) are a symptom of the prodromal phase of neurodegenerative disorders that are mechanistically linked to the protein α-synuclein (α-syn) including Parkinson’s disease (PD). SDs during the prodromal phase could result from neurodegeneration induced in state-controlling neurons by accumulation of α-syn predominant early in the disease, and consistent with this, we reported the monomeric form of α-syn (monomeric α-syn; α-syn_M_) caused cell death in the laterodorsal tegmental nucleus (LDT), which controls arousal as well as the sleep and wakefulness state. However, we only examined the male LDT, and since sex is considered a risk factor for the development of α-syn-related diseases including prodromal SDs, the possibility exists of sex-based differences in α-syn_M_ effects. Accordingly, we examined the hypothesis that α-syn_M_ exerts differential effects on membrane excitability, intracellular calcium, and cell viability in the LDT of females compared to males.

**Methods:**

Patch clamp electrophysiology, bulk load calcium imaging, and cell death histochemistry were used in LDT brain slices to monitor responses to α-syn_M_ and effects of GABA receptor acting agents.

**Results:**

Consistent with our hypothesis, we found differing effects of α-syn_M_ on female LDT neurons when compared to male. In females, α-syn_M_ induced a decrease in membrane excitability and heightened reductions in intracellular calcium, which were reliant on functional inhibitory acid transmission, as well as decreased the amplitude and frequency of spontaneous excitatory postsynaptic currents (sEPSCs) with a concurrent reduction in action potential firing rate. Cell viability studies showed higher α-syn_M_-mediated neurodegeneration in males compared to females that depended on inhibitory amino acid transmission. Further, presence of GABA receptor agonists was associated with reduced cell death in males.

**Conclusions:**

When taken together, we conclude that α-syn_M_ induces a sex-dependent effect on LDT neurons involving a GABA receptor-mediated mechanism that is neuroprotective. Understanding the potential sex differences in neurodegenerative processes, especially those occurring early in the disease, could enable implementation of sex-based strategies to identify prodromal PD cases, and promote efforts to illuminate new directions for tailored treatment and management of PD.

## Background

Parkinson’s disease (PD) is among one of the most widespread neurodegenerative disorders [1, 2], and sex is a risk-factor in the development of this disease, as PD is more common in men than in women with an approximated odds ratio of 2:1 [3–7]. Although PD is clinically diagnosed by the cardinal motor symptoms, evidence emerging over the past two decades has established sleep disorders (SDs) such as REM sleep behavior disorder (RBD), which is a sleeping disorder characterized by excessive motor behavior during what is normally a period of atonia, and excessive daytime sleepiness (EDS) as markers of the prodromal phase of PD, and these SDs can precede the motor symptoms by years to decades [8–13]. Sex has been acknowledged as an important determinant of both the susceptibility to neurodegenerative diseases and whether SDs co-occur following diagnosis, and while not well studied, it is likely that sex differences are also present in the prodromal phase of the disease prior to diagnosis, which could include the expression of SDs.

We hypothesized that the appearance of SDs prodromal to the motor symptoms in PD could be due to alteration of cellular function and neurodegeneration in sleep controlling nuclei and that sex differences in cellular effects could be present. Neurodegeneration in several brain nuclei in PD is associated with aggregation of the protein α-synuclein (α-syn), which is the histological hallmark of PD [14]. Pathological studies have shown that aggregated α-syn can interfere with several cellular functions in addition to promoting cell toxicity [15]. Heightened cell death has been reported in patients with α-synucleinopathies in the laterodorsal tegmentum (LDT) [16], which is a heterogenous nucleus comprised of cholinergic, glutamatergic and GABAergic neurons [17] that are importantly involved in the control of motor atonia during sleep, and arousal during wakefulness [18, 19]. Further, the co-occurrence of RBD and α-syn pathology was strongly correlated with brainstem cholinergic dysfunction in a predominantly male cohort consistent with α-syn-mediated degeneration of LDT cholinergic systems underlying aberrant behavioral state behavior [16]. Taken together, this suggests that α-syn-mediated PD processes include cellular actions in SD-controlling neurons already from abnormal levels of α-syn.

Although several studies have investigated the neuronal effects induced by α-syn, their focus was on actions of forms of the protein which aggregate (oligomeric and fibril). These forms are suspected to be the most damaging to neurons and neural transmission, and so very few studies have reported on cellular effects induced by the disordered, native monomeric form (α-syn_M_), which is widely considered to be relatively benign. The aggregation and fibrillation of α-syn may be preceded by dysregulation of expression e.g. as a result of SNCA gene multiplication [20]) and/or clearance [21] leading to abnormal levels of α-syn in the CNS, which then in turn may lead to nucleation and fibril formation [22]. We recently found that α-syn_M_ has cellular effects on neurons in the LDT that were associated with heightened cell death, which we speculated could play a role in SDs prodromal to, as well as following, diagnosis of PD. Specifically, we found that α-syn in monomeric form induced excitation, increased intracellular calcium, and heightened neuronal death of neurons of the LDT. In contrast,  different effects were induced in the substantia nigra (SN) in that α-syn_M_ elicited membrane inhibition, and greater decreases of intracellular calcium with no evidence of α-syn_M_-induced cell death [23]. However, that investigation was conducted solely in LDT of males. Because the appearance of sex-based differences in α-syn-related disease symptoms, including differences between SDs, suggest different mechanistic actions are involved in disease processes in males and females, we wished to determine whether excitatory cellular effects of α-syn_M_ on LDT, and heightened cell death in males were also present in females. Accordingly, in the present report, we have used electrophysiological and calcium imaging techniques ex vivo to investigate cellular effects of highly purified α-syn_M_ on LDT and SN neurons from females and compared effects to those in male LDT neurons.

## Methods

### Animals

The protocols for animal experiments used in this study were approved in concordance with the European Communities Council Directive (86/609/EEC). Brain slices of 250 μm thickness from female and male Naval Medical Research Institute (NMRI) mice aged 12 to 30 days (Harlan Mice Laboratories, Denmark) were used in electrophysiological, calcium imaging and cell viability studies. The animals were housed under the following conditions: temperature (22–23 °C), humidity (45–65%), light-dark cycle 12:12 h, water and food were available *ad libitum*.

### Brain slice preparations

A state of anesthesia was induced via inhalation of isoflurane (Baxter A/S, Denmark) and decapitation was conducted when anesthesia had been achieved as assayed by failure to react to a paw pinch. A block of the brain which contained LDT or SN was rapidly removed and submerged in ice-cold artificial cerebrospinal fluid (ACSF). The ACSF solution which contained 124 NaCl, 5 KCl, 1.2 Na_2_HPO_4_•2H_2_O, 2.7 CaCl_2_•2H_2_O, 1.2 MgSO_4_ (anhydrous), 10 dextrose, 26 NaHCO_3_ in mM was adjusted to a pH of 7.4 and an osmolarity of 298–302 mOsm/kg following saturation with carbogen (95% O_2_/5% CO_2_). The brain was sectioned in 250 μm thick slices containing the LDT or the SN with a vibratome (Leica VT1200S, Leica Biosystems, Germany). Brain slices were collected and placed in a chamber containing oxygenated ACSF, and incubated at 37 ^o^C for 15 min. To allow the tissue to equilibrate after the incubation period, the slices were kept at room temperature, and carbogen was continuously supplied for at least 1 h prior to further procedures, including exposure of the slice to the monomeric form of α-syn.

### Monomeric α-syn

Human α-syn was recombinantly expressed and purified as described in more detail in our earlier published work using this peptide [23]. Briefly, α-syn was cloned into *E. Coli* BL21DE3 cells using a pET-11a vector construct. Harvested cells were lysed by osmotic shock. Subsequently, boiling and centrifugation were conducted to remove non-heat-stable proteins. Ion-exchange chromatography was used to isolate α-syn, and the monomeric fraction was isolated by size exclusion chromatography SEC; thereafter, the monomers were pooled and kept in PBS buffer stored at -80^o^C until application to the slices.

### α-syn application

The monomeric form of α-syn (α-syn_M_) was stored in solution at -20^o^C in aliquots of 10 µl (150 µM) until use at which time it was applied via the bath. To reach a final concentration of 100 nM, an aliquot (150 µM) of 10 µl of α-syn_M_ was diluted in ACSF. After the establishment of baseline holding currents or baseline fluorescence, α-syn_M_ was applied for 3–4 min to monitor effects on membrane holding currents, synaptic activity, action potential firing, and intracellular calcium. For cell viability studies, incubation of the 250 μm brain slice in α-syn_M_ for 7 h was conducted in protocols described below.

### Drugs

Action potentials within the slice were blocked by 0.5 mM tetrodotoxin (TTX, Tocris, UK). Glycinergic receptors were blocked with strychnine (2.5 µM; Sigma, Denmark). GABA_A_ and GABA_B_ receptor-mediated responses were blocked by SR-95,531 (gabazine, 10 µM, Sigma, Demark) and CGP 55,845 (10 µM, Tocris, UK), respectively. Muscimol (30 µM, Sigma, Denmark) and baclofen (10 µM, Sigma, Denmark) were used as agonists of the GABA_A_ and GABA_B_ receptors, respectively. Stock solutions were stored in appropriate aliquots at -20^o^C prior and diluted in ACSF to final concentrations before use, and all drugs were applied via the bath.

### Patch-clamp recordings to monitor changes in membrane currents, synaptic activity, and action potential firing

Whole cell patch clamp recordings were conducted in 250 μm thick brain slices from neurons in the LDT (19 LDT brain slices; 14 mice) or the SN (7 SN brain slices; 6 mice). For recordings done in the LDT, we wished to target cholinergic neurons. Therefore, we selected the recorded neurons based on soma size (medium-to-large cells) and location within the central LDT wherein the concentration of cholinergic neurons is highest [24]. To fabricate patch pipette electrodes for recording neuronal electrical activity in LDT and SN brain slices, borosilicate filamented glass capillary tubes (1.5 mm, Sutter Instruments, USA) were pulled after heating to a sharp tip in a horizontal Flaming/Brown micropipette puller (P-97, Sutter Instruments, USA). These glass electrodes were filled with an intracellular solution (144 K-gluconate; 2 KCl; 10 HEPES; 0.2 EGTA; 5 Mg-ATP and 0.3 Na-GTP; in mM), which resulted in a pipette resistance of 6–11 MΩ. A brain slice containing LDT or SN was placed in the recording chamber that was situated in a microscope stage and ACSF saturated with a mixture of 95% oxygen/5% carbon dioxide (carbogen) was continuously perfused over the slice (flow rate 1.2 mL/min). A water immersion objective (60x) coupled to an upright microscope (BX50WI, Olympus; Japan) with an infrared Dodt gradient contrast system (IR-DGC; Luigs & Neumann, Germany) and a CCD camera (CCD-300ETRC; DAGE-MTI, Michigan City, IN) were used to visualize the cells. The software, Patchmaster (HEKA; version v2 × 91), was used to control a patch-clamp EPC9 amplifier (HEKA, Germany). Recordings were initiated in voltage-clamp mode to establish high resistance seals (> 1 MΩ) between the patch pipette and the cell membrane, and the holding voltage was kept at -60 mV. After at least a stabilization period of 7 min following membrane breakthrough, data were collected. AxoScope 10.2 (Molecular Devices Corporation, USA) and an Axon miniDigi 1B digitizer (Molecular Devices Corporation) were used to sample membrane effects. To quantify relative changes in holding currents, the holding currents in pA averaged from at least 30 sec of recording before application of α-syn_M_ and the holding currents averaged from at least 30 sec during the maximum effect of α-syn_M_ were subtracted. A change in amplitude of 2 pA from baseline was used as criteria to be considered a response. For firing frequency studies, action potentials were recorded in current-clamp mode before and after α-syn_M_ application. Current was applied to depolarize the neuron sufficiently to induce a sustained firing of action potentials (-45 mV). A period of firing in an epoch of 30 sec immediately prior to and 30 sec after application of α-syn_M_ was selected and interspike intervals were measured and averaged. Interspike intervals were determined by measuring the period of time (in msec) from the initiation of a spike to initiation of the next spike.

### Calcium imaging to monitor changes in intracellular calcium

Intracellular loading of cells with a calcium binding dye was performed in 42 LDT_F_ (25 mice) and 15 LDT_M_ (7 mice) 250 μm thick brain slices following standard protocols [25] for single-photon calcium imaging. Recordings were conducted utilizing ratiometric fluorescent calcium indicator dye Fura-2 acetoxymethyl ester (Fura-2 AM). Prior to recordings, slices were rinsed for over 15 min in the recording chamber by continuous perfusion of oxygenated ACSF at a flow rate of 1.2 ml/min to wash out free dye debris and allow temperature equilibration. To localize the LDT, an upright microscope (BX50WI, Olympus, Germany) was used under bright field illumination and visualization guided by characteristic landmarks located close to these nuclei. Individual cells were viewed with a water immersion objective (40x). A cooled CCD fluorescence camera (12-bit Sensicam, PCO Imaging, Germany) attached to the microscope and controlled by the imaging software Live Acquisition (TILL Photonics, Germany) was used to collect paired images that were binned at 2 × 2 pixels on the camera chip in order to optimize spatial and temporal resolution. Live Acquisition controlled rapid switching between the excitation wavelengths of 340 and 380 nm in order to ensure a minimal amount of time passing between collection of the image at each wavelength, which allowed ratiometric calculations. The acquisition interval between each frame pair of 340 and 380 nm was 4 sec. Regions of interest (ROI) were drawn around each Fura-2 loaded cell, as well as around a region of the field of view that contained no dye loaded cells, which was used to measure background fluorescence. Analysis software (Offline Analysis, TILL Photonics, Germany) was used to quantify fluorescence in each ROI by averaging the intensity of the 2 × 2 binned pixels, and background in each channel was subtracted. The 340 and 380 nm channels were ratioed (340 nm:380 nm). Baseline fluorescence was calculated from an average of intensity taken from 10 frames collected before drug application. Following application of α-syn_M_, changes in fluorescence from that measured at baseline were noted in the majority of cells, and the fluorescence of the peak deflection from baseline was measured by averaging across 10 frames. Data are presented as DF/F% which represents the subtraction of the baseline fluorescence from the maximum change in fluorescence induced by α-syn_M_ application divided by the baseline, followed by conversion to a percentage. Ascendent deflection of fluorescence indicates intracellular calcium elevations with descendent deflections reflecting decreases in intracellular calcium. Actual calcium levels were not calculated due to well-known complications with converting changes in fluorescence in brain slices to calcium concentrations [26, 27].

### Neurotoxicity assay to evaluate cell viability

Propidium iodide (PI; Sigma-Aldrich) was used to identify dead cells and 4′,6-diamidino-2-phenylindole (DAPI; Sigma-Aldrich) was used to mark live cells from LDT_F_ and LDT_M_ slices. A total of 116 slices from 56 mice were used in 3 different protocols in which 250 μm slices were bisected with each half being exposed for 7 h under oxygenation to: (1) ACSF or α-syn_M_ (100 nM), (2) α-syn_M_ (100 nM) or α-syn_M_ (100 nM) plus GABA_A_, GABA_B_ and glycine receptor antagonists, or (3) α-syn_M_ (100 nM) or α-syn_M_ (100 nM) plus GABA_A_, GABA_B_, and glycine receptor agonists. The bisection of the slices ensured that data sets could be compared from tissue taken from the same group of animals, and that protocols were run side by side under the same laboratory conditions. Following incubation, slices were fixed in 4% paraformaldehyde overnight, cryprotected with sucrose saturation (30%), and resectioned to a thickness of 40 μm on a cryostat (Leica CM3050, Triolab, DK). Resectioned slices of 40 μm were incubated for 3 periods of 5 min in a solution which contained 1 µg/ml of both PI and DAPI with a pH of 7.4. To detect fluorescence signals from PI and DAPI, an upright Zeiss microscope coupled to a monochrome CCD camera (Axiocam MRM, Zeiss, Germany) controlled by Axioskop 2 software (AxioVision 4.6, Zeiss) and required filter cubes were used (Zeiss 59 fluorescent filter cube sets, wavelengths PI: 472–578 nm; DAPI: 358–463 nm).

To conduct analysis of collected images, a macro written for ImageJ (National Institutes of Health, Bethesda, MD) was used to automatically count the number of DAPI and PI-labeled cells following background subtraction, application of thresholding, and separation of objects via a watershed algorithm. Cells were selected using the batch processing macro, Analyze Particle; however, selections were manually confirmed. Cell survival was quantified by the proportion of live cells (DAPI positive cells) to the total cell count, which was calculated as the addition of PI positive cells and DAPI positive cells. Cell viability was evaluated in up to 5 areas within the LDT in the 40 μm resectioned slices. For this data set, n_a_ = the number of areas examined/n_40_ = the number of resectioned slices. Data was normalized in each of the 3 protocols to the control condition, which was either exposure to ACSF or α-syn_M_ without GABA_A_, GABA_B_, and glycine receptor agonists or antagonists. However, to compare cell death between LDT_F_ and LDT_M_, cell survival was evaluated without normalization. In figure panels depicting PI positive and DAPI positive cells, contrast has been applied equally across entire images.

### Data analysis and statistics

Amplitudes of membrane holding currents were measured (the difference between baseline and maximum deflection) using Axoscope 10.5 (Molecular Devices, USA). Spontaneous excitatory synaptic events (sEPSCs) were detected and analyzed using MiniAnalysis (Synaptosoft, USA). Analysis of synaptic events was conducted by selecting 30 sec of the recording just before application of α-syn_M_ and at the peak amplitude of the effect on membrane current. Inter-event intervals and the amplitude of events were averaged across a population of cells and statistically analyzed. Firing frequency was analyzed selecting 30 sec epochs before and after α-syn_M_ application, and intervals between action potentials were measured and averaged. Calcium imaging data including the amplitudes of DF/F% changes were analyzed in Graphpad Prism (version 7.0). The numbers of observations in the data sets analyzed for cell viability are reflected as n_a_. Results are presented as mean values ± SEM. The figures were prepared using Igor Pro software (Wavemetrics, USA) and GraphPad Prism. Differences in numerical data were tested using a Paired or Unpaired Student’s T-test, and differences in categorical data were examined using the Fisher’s Exact test. P values are reported in text as 4 decimal points, and a significant difference was determined if alpha was less than 0.05.

## Results

### **α-syn**_**M**_ **effects on membrane currents, and synaptic transmission in neurons of sleep and motor controlling nuclei in the female**

#### α-syn_M_ induced outward currents in sleep and motor control nuclei in the female LDT

**Fig. 1:**
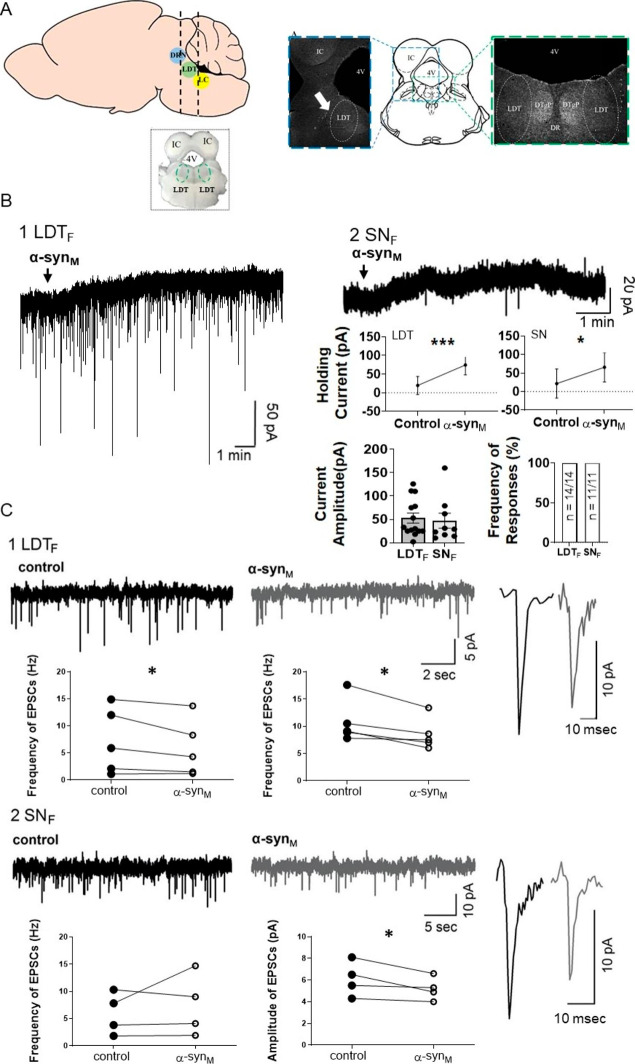
α-syn_M_ induced an inhibitory outward current and modulated synaptic transmission in LDT neurons in the female. **A)** (Left) Cartoon schematic of the sagittal mouse brain to show the block of the brain containing the LDT. A LDT coronal brain slice taken from this block is shown below in inset. **(A)** (Right) Coronal brain slice cartoon modified from [72] to show in greater detail in insets to the sides the location of the LDT (indicated by white arrow in panel to the left). **(B)** Sample of membrane response to α-syn_M_, which induced inhibitory, outward currents in the female in the LDT **(B1)**. An outward inhibitory current was also elicited in SN neurons **(B2)**. **(B)** Graphs of holding currents before and after application of α-syn_M_ to LDT_F_ and SN_F_ neurons showed a significant increase in positive holding current indicating that α-syn_M_ induced outward currents. The amplitude of the outward current evoked by α-syn_M_ in LDT_F_ and SN_F_ neurons was not different (LDT_F_: n = 14, SN_F_: n = 11; p = 0.3847; Unpaired Student’s T-test) as shown by the plots of the individual amplitude of current induced in both nuclei. Bar chart showing that the proportion of recorded cells responding to α-syn_M_ with induction of outward current did not differ significantly between the LDT_F_ and SN_F_ (LDT_F_: n = 14 sampled/14 responded, SN_F_: n = 11 sampled/11 responded; p = 1.000; Fisher’s Exact Test). **(C)** α-syn_M_ modulated synaptic events in neurons recorded within LDT_F_ and SN_F_. **(C1- C2)** samples of recordings showing frequency of synaptic events in control and in presence of α-syn_M_ in both LDT_F_ and SN_F_. (Rightmost panels) Single sEPSCs (spontaneous excitatory postsynaptic currents) in a LDT_F_ and in a SN_F_ neuron are shown with a high-gain time and amplitude scale under control conditions and in presence of α-syn_M_ illustrating the reduction in amplitude in both nuclei when α-syn_M_ was present. Data presented in paired plots summarize findings from the population of recorded cells, which revealed that α-syn_M_ induced a significant decrease in amplitude of EPSCs in LDT_F_ (n = 5; p = 0.0253; Paired T-test) and SN_F_ neurons (n = 4; p = 0.0483; Paired T-test) and elicited a significant decrease in the frequency of sEPSCs in LDT_F_ neurons (n = 5; p = 0.0475; Paired T-test), which was a change not seen in sEPSCs in the SN (n = 4; p = 0.4735; Paired T-test). LDT: Laterodorsal tegmental nucleus; 4 V: 4th ventricle; IC: Inferior colliculus; DTgP: Dorsal tegmental nucleus, pericentral: DRN: dorsal raphe nucleus; LC: Locus coeruleus; LDT_F_: Laterodorsal tegmental nucleus of female; SN_F_: Substantia nigra of female. * Indicates p < 0.05, *** Indicates p < 0.001

Our previous study showed that α-syn_M_ induced an inward current in LDT neurons in brain slices from male mice (LDT_M_) [23]. Unexpectedly, in neurons in LDT brain slices from female mice (LDT_F_; Fig. 1A), α-syn_M_ (100 nM, 3 min) induced an outward membrane current in all cells examined (amplitude: 54.8 ± 11.3 pA, n = 14). To compare the effect of α-syn_M_ in neurons of a sleep controlling nucleus vs. neurons of a motor control nucleus, we next investigated the neuronal effect of α-syn_M_ in the SN of females (SN_F_). Similar to our previous report that showed α-syn_M_ induced inhibitory membrane responses in neurons of the male SN (SN_M_), we observed that α-syn_M_ induced inhibitory responses in the membrane of 100% of the neurons examined in SN_F_ (amplitude: 47.2 ± 16.0 pA, n = 11). The average amplitude of the outward current induced in neurons of the LDT_F_ did not differ from that induced in SN_F_ neurons (p = 0.3847; Unpaired Student’s T-test; Fig. 1B).

#### α-syn_M_ alters synaptic events in sleep and motor control nuclei in the female LDT

Our previous study showed that α-syn_M_ altered synaptic transmission in LDT_M_ neurons producing increases in frequency as well as amplitude of spontaneous excitatory postsynaptic currents (sEPSCs). However, in the present study, the opposite effect was seen in LDT_F_ as α-syn_M_ induced a significant decrease of nearly 20% in sEPSC frequency (Control: 7.2 ± 2.7 Hz; α-syn_M_: 5.8 ± 2.3 Hz; p = 0.0475; n = 5, Paired T-test) and a 21% decrease in the amplitude of sEPSCs was noted which was significant (control: 10.8 ± 1.7 pA; α-syn_M_: 8.5 ± 1.3 pA; n = 5; p = 0.0253; Paired T-test; Fig. 1C).

In SN_F_, α-syn_M_ induced a decrease of 14% in amplitude of sEPSCs which was significant when compared to control (control: 6.1 ± 0.8pA; α-syn_M_: 5.2 ± 0.5pA; n = 4; p = 0.0483; Paired T-test); however, changes induced in the frequency were not significantly different (control: 5.9 ± 1.9 Hz; α-syn_M_: 7.4 ± 2.8 Hz; n = 4; p = 0.4735; Paired T-test; Fig. 1C). In our previous work, we did not examine the effect of α-syn_M_ on sEPSCs in the SN in the male (SN_M_). Therefore, in order to examine sex-based potential differences of α-syn_M_ on sEPSCs in this motor control nucleus, we determined whether α-syn_M_ had effects on synaptic activity in the SN_M_ and found that changes in sEPSCs were qualitatively similar to those seen in SN_F_. α-syn_M_ induced a decrease of 10% in the amplitude of the current of the sEPSCs (Ctrl: 6.0 ± 1.7 pA; α-syn_M_: 5.4 ± 1.6 pA; n = 4; p = 0.0449; Paired T-test), and changes induced in the frequency were not significantly different (Ctrl: 11.8 ± 2.2 Hz; α-syn_M_: 11.7 ± 2.4 Hz; n = 4; p = 0.9162; Paired T-test). In summary, these data indicate that α-syn_M_ induced inhibitory membrane effects on neurons of LDT_F_, and a reduction in the amplitude and frequency of sEPSCs, which were opposite effects from those we have reported before in LDT_M_ [23]. In SN_F_, α-syn_M_ also induced inhibitory effects on the membrane, which were similar to those effects we have reported before in SN_M_. Further, α-syn_M_ had similar effects on synaptic events in SN_M_ to those seen in SN_F_ as in both sexes we observed a reduction in the amplitude of sEPSCs with no effect on frequency. Taken together, our findings show that the examined effects of α-syn_M_ on LDT neurons are sex-dependent, whereas, α-syn_M_ effects on SN are independent of sex.

### Sex differences in LDT neurons of α-syn_M_-mediated alteration of intracellular calcium

**Fig. 2:**
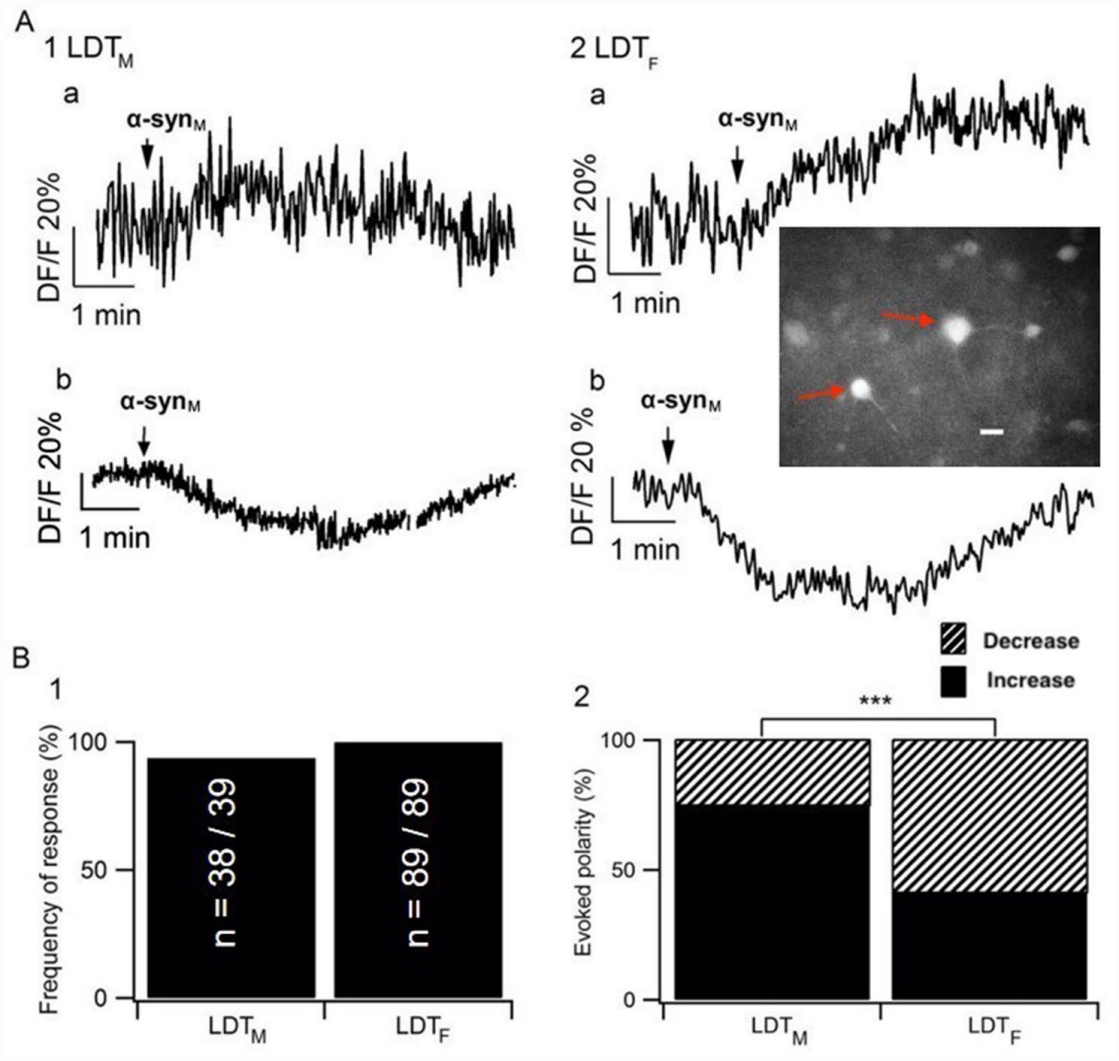
Sample of changes in fluorescence (DF/F%) induced by α-syn_M_, which are indicative of alterations in intracellular calcium levels in LDT_M_ and LDT_F_. **(A)** In both sexes, changes in response of the fluorescence to α-syn_M_ exhibited two different polarities, which suggested increases **(A1a, A2a)** or decreases **(A1b, A2b)** in intracellular calcium levels, respectively. Inset in A2 is a fluorescent image under 380 nm wavelength light of one of the LDT_F_ brain slices used in this study in which two Fura 2-AM filled cells indicated with red arrows can be seen. Regions of interest were drawn around each cell and average fluorescent intensity (F) within each region of interest was plotted against time. White scale bar indicates 20 μm. **(B)** Histograms summarizing the data from the population of recorded cells indicating that **(B1)** the frequency of responses to α-syn_M_ did not significantly differ between the two sexes (LDT_M_: n = 38/39, LDT_F_: n = 89/89; p = 0.3047; Fischer’s Exact test), **(B2)** whereas the distribution of response polarity differed significantly between the sexes with a greater proportion of responses suggesting decreases in calcium being elicited in females than males (LDT_M_: n of decreases = 9/38, LDT_F_: n of decreases = 52/89; p = 0.0004; Fisher’s Exact test). LDT_M_: Laterodorsal tegmental nucleus of male; LDT_F_: Laterodorsal tegmental nucleus of female. *** Indicates p < 0.001

As α-syn_M_-induced neuronal effects have been hypothesized to lead to calcium dysregulation [23, 28], we previously examined actions of this protein on intracellular calcium levels and found that α-syn_M_ induced changes in calcium in LDT_M_. We repeated those experiments here and confirmed our earlier findings. Using multiple-cell calcium imaging to monitor changes in Fura 2-AM fluorescence which were induced by α-syn_M_ (100 nM, 3 min), we observed changes in fluorescence in the majority of LDT_M_ cells (97.4%; n = 38/39; Fig. 2B1), and the majority of responses were indicative of an increase in calcium (76.3%; n = 29/38; Fig. 2A1a, B2). We then examined responses in LDT_F_ and found that α-syn_M_ induced changes in intracellular calcium in 100% (n = 89/89) of the examined cells (Fig. 2B1). However, interestingly, the majority of responding LDT_F_ neurons exhibited changes in fluorescence indicative of a decrease in intracellular calcium levels (58.4%, n = 52/89; Fig. 2A2b, B2). When we compared alterations in intracellular calcium induced by α-syn_M_ in LDT_M_ to LDT_F_, there was no significant difference in the numbers of responding or non-responding neurons (p = 0.3047; Fischer’s Exact test; Fig. 2B1). In contrast, there was a difference between males and females in the ratio of increases in calcium to decreases in calcium in response to α-syn_M_. We observed a significantly higher proportion of cells responding with a decrease in calcium in LDT_F_ when compared to the proportion of cells exhibiting decreases in LDT_M_ (p = 0.0004; Fisher’s Exact test; Fig. 2B2).

### **Evaluation of the mechanism of α-syn**_M_**inhibition induced in the membrane of LDT neurons**

**Fig. 3:**
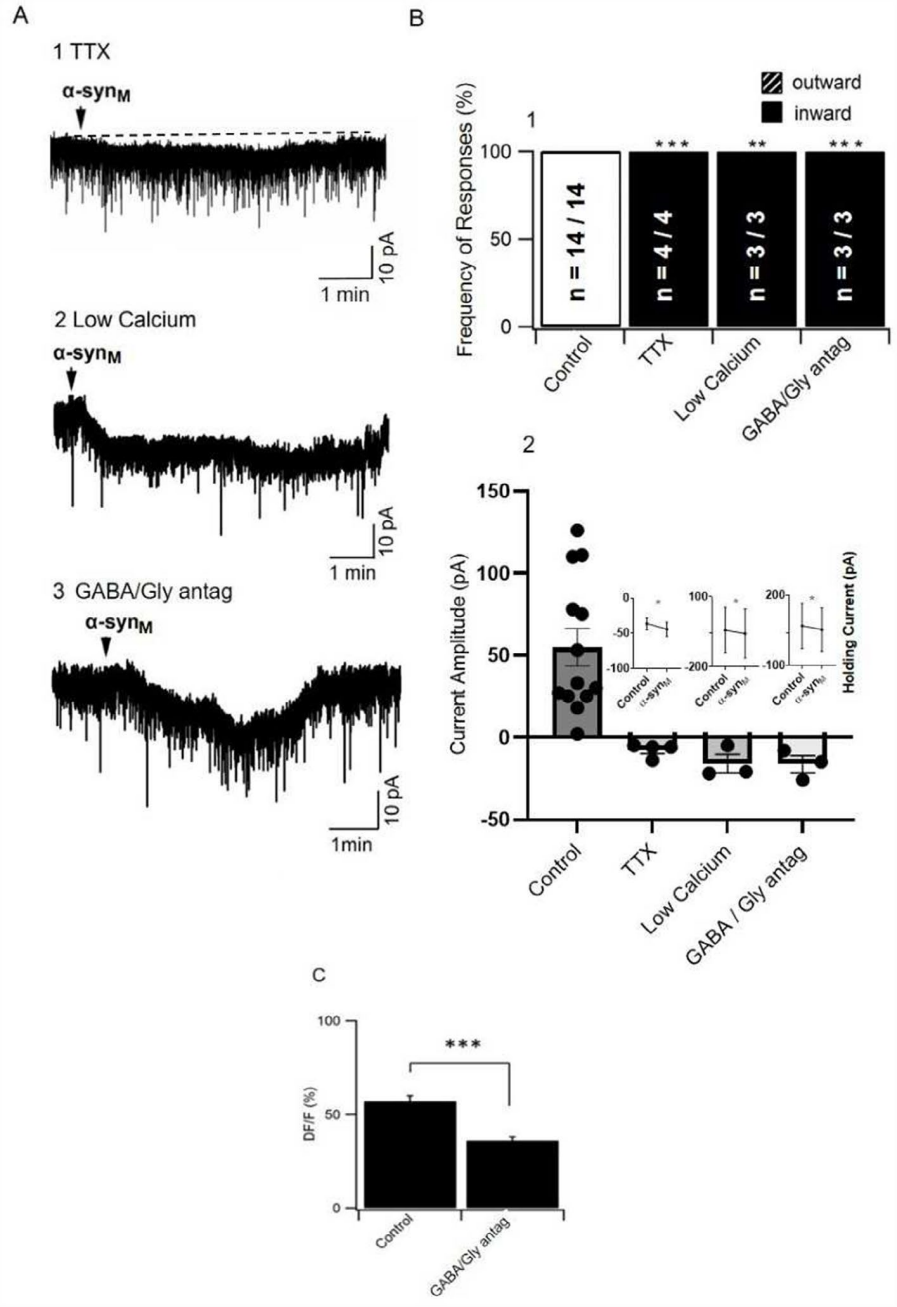
An excitatory inward current was revealed when α-syn_M_ was applied during blockade of presynaptic transmission, or antagonism of GABA_A_, GABA_B_ and glycine receptors, suggesting that α-syn_M_ induces an outward membrane current in LDT_F_ neurons due to actions at presynaptic inhibitory neurons. **A**) Sample of membrane responses to α-syn_M_ in which an inward current is revealed when α-syn_M_ is applied in presence of **(A1)** TTX, **(A2)** low calcium solution, or **(A3)** a cocktail of SR-95,531 + CGP-55,845 + strychnine, which block GABA_A_, GABA_B_ and glycine receptors, respectively). **(B)** Histograms from a population of cells recorded in which there were significant changes in the **(B1)** polarity of the evoked membrane current responses to α-syn_M_ in presence of TTX, low calcium solution or GABA and glycine receptor antagonists when compared to control responses (Fisher’s Exact test). **(B2)** The amplitude of the current evoked by α-syn_M_ in control conditions and under conditions of synaptic blockade and inhibitory receptor antagonists is shown revealing the change in polarity of the α-syn_M_ induced current. **(B2, Inset)** Graphs of holding currents before and after application of α-syn_M_ to LDT_F_ showed that in presence of TTX, low calcium solution, and GABA_A_, GABA_B_, and glycine receptor antagonists, holding currents became significantly more negative after application of α-syn_M_, which reflected the α-syn_M_-mediated induction of inward currents. **(C)** The decrease in intracellular calcium in LDT_F_ is mediated, at least in part, by inhibitory receptors as illustrated in this histogram showing data from the population of LDT_F_ cells recorded that showed a significantly smaller decrease in the amplitude of intracellular calcium induced by α-syn_M_ in presence of SR-95,531, CGP-55,845 and strychnine when compared to control conditions (Control: n = 52, GABA/Gly antagonists: n = 49; p = 0.0001; Paired T-test). LDT_F_: Laterodorsal tegmental nucleus of female. * Indicates p < 0.05, ** Indicates p < 0.01, *** Indicates p < 0.001

To gain more information regarding the sex-based difference in the mechanism underlying α-syn_M_ membrane effects, we examined α-syn_M_ actions during blockade of the generation of Na^+^-dependent action potentials by inclusion in the bath of tetrodotoxin (TTX, 500 nM). Unexpectedly, in presence of TTX, α-syn_M_ induced an inward current (-7.7 ± 2.0 pA, n = 4), and this effect was present in all neurons tested (Fig. 3A, B).

Our findings with TTX indicated that α-syn_M_-induced outward currents relied on a presynaptic mechanism. To confirm the involvement of a presynaptically-mediated mechanism in the induction of outward currents in the postsynaptic membrane of LDT_F_ neurons, we next applied α-syn_M_ in a reduced calcium solution, which effectively eliminates calcium-dependent synaptic transmission. Because we wished to use a within cell control, we first verified whether a second application of α-syn_M_ to the same cell could result in similar effects on the membrane as those elicited in a first application. Thus, in a subset of LDT_F_ neurons, we reapplied α-syn_M_ following a first application, and we observed that a second inhibitory response was elicited in all neurons tested, which did not significantly vary in amplitude from the outward current obtained in the first application (n = 3). Consistent with the TTX data, in all the cells tested in which the first application elicited an outward current, in the second application in presence of low calcium solution, we did not observe an induction of an outward current, and instead, an inward current was elicited (-16.1 ± 5.5 pA; n = 3; Fig. 3A2, B). Taken together, these data indicate that the inhibitory effect induced by α-syn_M_ on the membrane of LDT_F_ involves presynaptic-dependent mechanisms.

LDT neurons receive a heavy inhibitory input from presynaptic GABAergic terminals both from local LDT neurons but also from projections sourcing from outside the nucleus [29], and, accordingly, GABAergic presynaptic mechanisms could be involved in α-syn_M_-mediated inhibitory responses in LDT_F_. Therefore, we investigated α-syn_M_-induced membrane responses in LDT_F_ in presence of the GABA receptor antagonists, SR-95,531 (10 µM) and CGP-55,845 (10 µM), which block GABA_A_ and GABA_B_ receptors, respectively. Although no evidence has been presented of glycine-mediated inhibition of LDT cells, and we have not noted any glycinergically-mediated spontaneous inhibitory currents (sIPSCs) in our own studies under our recording conditions, we also included strychnine (2.5 µM) in the ACSF to ensure the blockade of any glycine-mediated events. In a population of cells in which the first application of α-syn_M_ induced an outward current, we found that in the presence of GABA and glycine receptor blockade, an inward current was elicited in all tested cells (-16.3 ± 5.2 pA; n = 3; Fig. 3A3, B1, 2).

When taken together, our data indicate that induction of outward current in LDT_F_ neurons is reliant on inhibitory transmission from presynaptic neurons. Blockade of inhibitory transmission revealed an inward membrane current similar to what has been seen in LDT_M_. Although not tested in the present study, we showed in our previous work that inward currents in LDT_M_ were mediated by a G-protein receptor coupled mechanism in postsynaptic neurons. Although we conducted experiments to examine a role for receptors previously implicated in α-syn_M_ effects, we could not identify the specific receptor involved; however, we speculate that this same excitatory mechanism is being activated in in LDT_F_ but masked by the concurrent induction of outward current induced by α-syn_M_-mediated stimulation of inhibitory presynaptic transmission.

#### Inhibitory amino acids are involved in the decrease of intracellular calcium

As we had seen that membrane current effects of α-syn_M_ involved inhibitory transmission, we examined whether similar mechanisms were also involved in the decrease of intracellular calcium seen in response to α-syn_M_ in the majority of neurons of the LDT_F_. During blockade of GABA and glycine receptors, while decreases in fluorescence indicative of reductions in calcium were still elicited, the amplitude of the decrease in fluorescence was significantly smaller (36%) compared to that elicited in control conditions (control: 57.1 ± 3.0% DF/F, n = 52; blockers: 36.2 ± 2.3% DF/F, n = 49; p = 0.0001; Paired T-test; Fig. 3C). Taken together, while they suggest that other mechanisms might be involved in the reductions in intracellular calcium induced by α-syn_M_, these data provide evidence that inhibitory amino acids, most likely GABA contribute to α-syn_M_-mediated calcium decreases in LDT_F_ and provide further support that inhibitory transmission targeting postsynaptic LDT cells is activated by α-syn_M_.

### α-syn_M_ Reduces the excitability of LDT neurons in the female

**Fig. 4:**
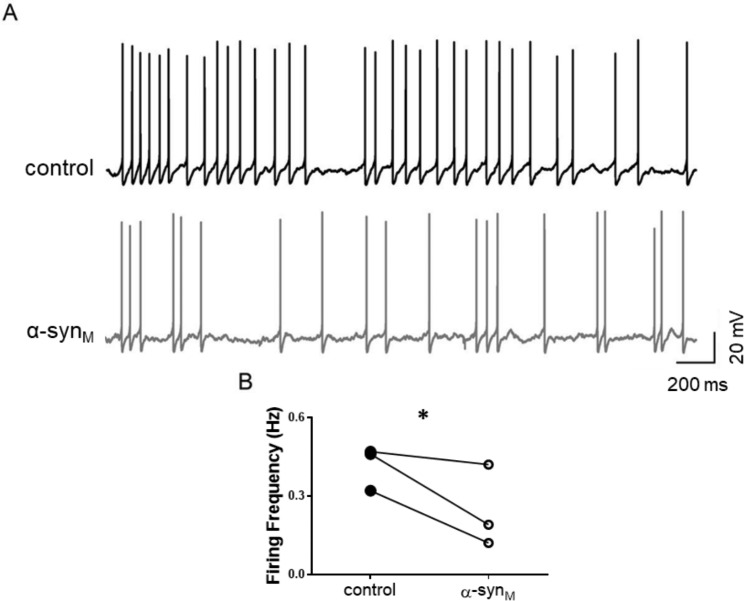
α-syn_M_ induces significant changes in the firing frequency in neurons recorded within LDT_F_. **(A)** Representative examples of current-clamp recordings of LDT neurons in which action potentials were induced by holding the cell at -45 mV under control conditions (top) and in presence of α-syn_M_. (bottom). **(B)** The reduction in firing frequency induced by α-syn_M_ was significant as shown in the bar graphs depicting the average firing rate from the population of recorded LDT_F_ neurons (n = 3; p = 0.0498; Paired T-test). LDT_F_: Laterodorsal tegmental nucleus of female * Indicates p < 0.05

We previously reported that α-syn_M_ enhanced the firing frequency of neurons within LDT_M_. However, the inhibitory effect induced by α-syn_M_ on the membrane of LDT_F_ in conjunction with the reduction in amplitude and frequency of EPSCs could reduce neuronal excitability in the female. To directly investigate the functional effect of α-syn_M_-mediated actions on LDT_F_ neurons which could affect the output of these cells, we examined the firing frequency in current clamp mode following depolarization of the membrane of LDT_F_ neurons sufficiently to induce action potentials (V_M_: -45.0 ± 5.0 mV) before and after application of α-syn_M_. Under these conditions, α-syn_M_ reduced the firing frequency by 36.5% from baseline levels (control: 0.41 ± 0.05 Hz; α-syn_M_: 0.26 ± 0.08 Hz; n = 3; p = 0.0498; Paired T-test; Fig. 4A, B). These data suggest that in direct contrast to findings in LDT_M_, functional actions of α-syn_M_ include reductions in neuronal excitability of neurons in LDT_F_, which would be expected to alter output of these cells to target regions.

### **α-syn**_M_**induces a lower cell death of LDT neurons in females compared to males**

**Fig. 5:**
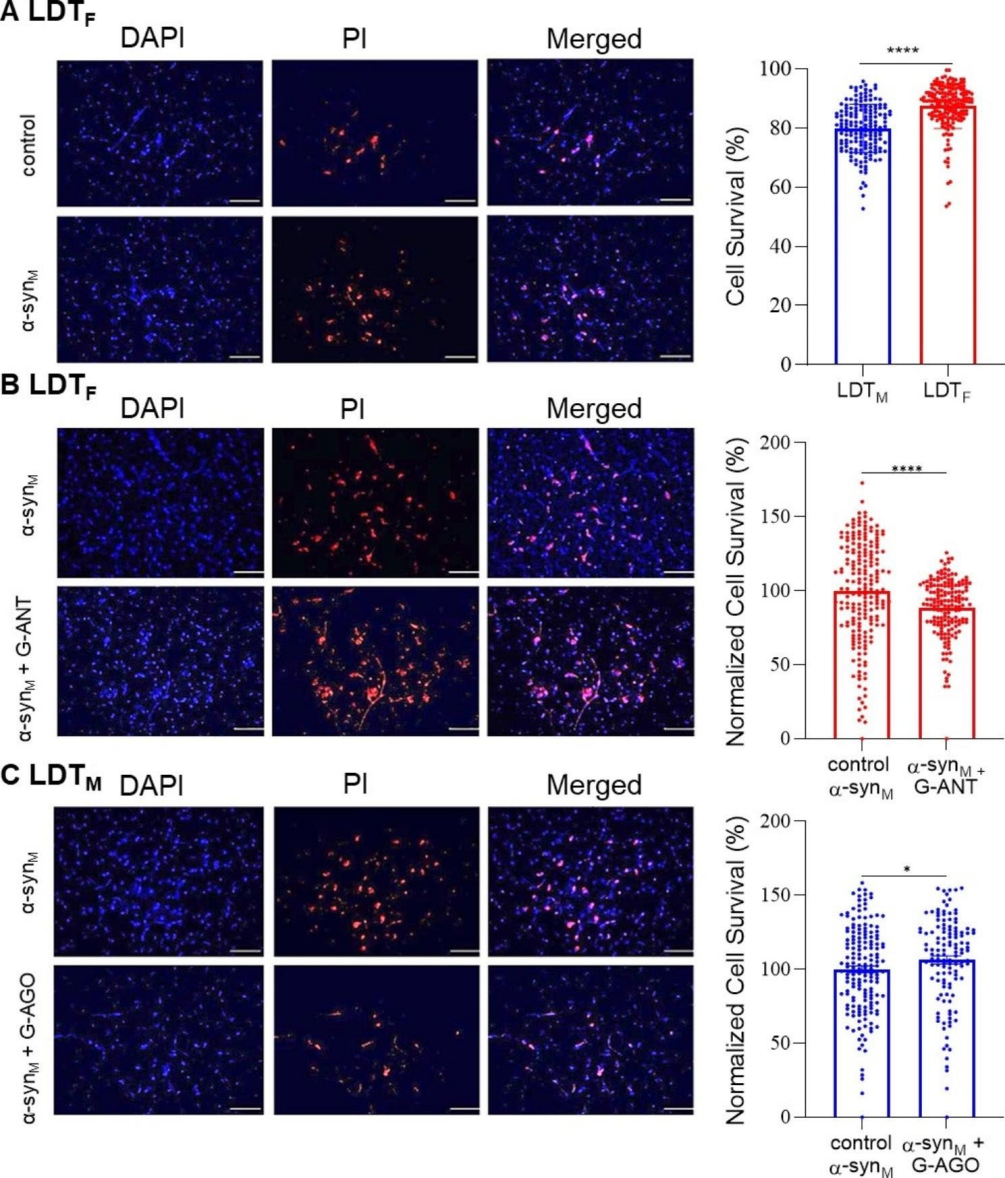
**(A-C, left panels)** DAPI and PI immunohistochemistry conducted in LDT_F_ and LDT_M_ from brain slices exposed to α-syn_M_ under 3 different treatment protocols is shown in representative fluorescent images. The first column represents living cells visualized by DAPI (blue). The second column represents dead cells visualized by PI (red), and the last column is a merged image of DAPI and PI labeled cells. **(A)** The presence of DAPI and PI in the LDT_F_ following incubation of one half of a slice in control solution (ACSF) and the other half in ACSF containing α-syn_M_ for 7 h is shown and indicates relatively lower cell survival in the half of the slice exposed to α-syn_M_, which was reflected in the population data. The bar graph to the right shows that following α-syn_M_ exposure, cell survival in LDT_F_ was significantly greater than that seen in LDT_M_ (Cell Survival Female: n_a_ = 183/n_40_ = 48, Cell Survival Male: n_a_=168/n_40_ = 66; p < 0.0001; Unpaired Student’s T-test). In this and subsequent panels, red points represent observations from LDT_F_, blue represent data from LDT_M_, and cell counts within each area (n_a_) represent one data point or observation in the bar chart columns. **(B)** Fluorescent images showing DAPI and PI presence in LDT_F_ cells treated for 7 h with α-syn_M_ or with α-syn_M_ in presence of GABA_A_, GABA_B_ and glycine receptors antagonists (G-ANT). As shown in the bar graph to the right, a reduced cell survival indicative of greater cell mortality was observed in the population of LDT_F_ slices exposed to α-syn_M_ when GABA and glycine receptor antagonists were present (Cell survival: α-syn_M_: n_a_ = 199/n_40_ = 44, Cell survival α-syn_M_ + G-ANT: n_a_ = 169/n_40_ = 35; p = 0.0001; Mann-Whitney Test). To compare the population data, bisected slices were used, and the proportion of surviving cells observed in the half of the bisected slice exposed to α-syn_M_ was considered the baseline, and the number of surviving cells in the other half of the bisected slice exposed to α-syn_M_ + G-ANT was normalized to this baseline. **(C)** Fluorescent images of LDT_M_ slices exposed to ⍺-syn_M_ or to α-syn_M_ in presence of 7 h of GABA_A_ and GABA_B_ receptor agonists (G-AGO). As can be seen from the population data shown in bar graphs to the right, the presence of the GABA and glycine receptor agonists in the LDT_M_ was associated with significantly greater cell survival following exposure to α-syn_M_ (Cell Survival α-syn_M_: n_a_ = 168/n_40_ = 66, Cell Survival α-syn_M_ + G-AGO: n_a_ = 127 /n_40_ = 48; p = 0.0334; Mann-Whitney Test). In this protocol, the proportion of surviving cells observed in the half of the bisected slice exposed to α-syn_M_ was considered the baseline, and the number of surviving cells in the other half of the bisected slice exposed to α-syn_M_ + G-AGO was normalized to this baseline. LDT_F_: the laterodorsal tegmental nucleus of female; LDT_M_: the laterodorsal tegmental nucleus of male. G-ANT: contains SR-95,531 (gabazine, 10 µM), CGP 55,845 (10 µM) and strychnine (2.5 µM) to block GABA_A_, GABA_B_, and glycine receptor-mediated responses, respectively. G-AGO: contains muscimol (30 µM) and baclofen (10 µM), which are agonists of GABA_A_ and GABA_B_ receptors, respectively. The scale bar in all images corresponds to 50 μm under 40x magnification. Contrast has been added equally across all the images. *p < 0.05, **** p < 0.0001

In our previous report, α-syn_M_-mediated excitation of the membrane of neurons with a concurrent rise of intracellular calcium was suspected to induce excitotoxicity, which was supported by a heightened cell death over control in LDT_M_ [34]. As α-syn_M_-induced inhibitory membrane current, and increases in calcium were less prominent in LDT_F_ neurons, we hypothesized that neurodegeneration induced by α-syn_M_ would also exhibit a sex-based difference. First, we needed to determine whether α-syn_M_ induced cell death in LDT_F_ above control. Accordingly, we evaluated cell survival in LDT_F_ hemi slices in which one half had been exposed for 7 h to ACSF and the other half to 7 h in α-syn_M_. We found a relatively lower cell survival in the half of the slice exposed to α-syn_M_ when normalized to survival seen in control (Cell Survival: Control: ACSF: 100 ± 0.9%, n_a_ = 217/n_40_ = 57; α-syn_M_: 92.8 ± 1.5%, n_a_ = 183/n_40_ = 48; Fig. 5A).

Next, we compared cell survival in the halves of the LDT_F_ exposed to α-syn_M_ for 7 h to halves of brain slices of the LDT_M_ that had been similarly exposed to 7 h of α-syn_M_. Supporting our hypothesis of a sex difference in α-syn_M_ cellular effects, we noted a significantly greater cell survival in the LDT_F_ when compared to cell survival seen in the LDT_M_ (Cell Survival: Female: 87.4 ± 0.6%, n_a_ = 183/n_40_ = 48; Males: 79.9 ± 0.6%, n_a_ = 168/n_40_ = 66, p < 0.0001; Unpaired Student’s T-test; Fig. 5A). These results indicate that α-syn_M_ induces toxic effects in neurons of the LDT_F_ to a lesser degree than in LDT_M_.

### Endogenous neuroprotection against α-syn_M_ induced degeneration in LDT neurons from female involved inhibitory transmission

As we showed a sex-based differential effect of α-syn_M_ on neuronal mortality in the LDT which was reminiscent of the sex-based difference in membrane excitability and changes in intracellular calcium levels induced by this protein which were affected by GABA and glycine receptor antagonists, we reasoned that the differential effect on neurodegeneration could involve inhibitory amino acid activity which was protective in the LDT_F_. To examine this hypothesis, we exposed LDT_F_ cells to α-syn_M_ for 7 h in the presence of antagonists of GABA_A_, GABA_B_ and glycine receptors (G-ANT) and normalized cell viability to that in the other halves of the bisected slices that were exposed only to α-syn_M_. The relative degree of LDT_F_ of cell survival associated with α-syn_M_ in the presence of G-ANT was significantly lower than in absence of blockers of receptors of inhibitory amino acids (Cell survival: α-syn_M_: 100.0 ± 2.4%, n_a_ = 199/n_40_ = 44, α-syn_M_ + G-ANT: 88.8 ± 1.5%, n_a_ = 169/n_40_ = 35; p = 0.0001; Mann-Whitney Test; Fig. 5B). These results indicate that the neuroprotective effects seen in the female brain against α-syn_M_-mediated toxicity involve functional inhibitory amino acid transmission.

### Activation of GABAergic transmission in male brain induces neuroprotection against α-syn_M_ -induced neurodegeneration

Next, we hypothesized that activation of GABA receptors could be neuroprotective against α-syn_M_-mediated toxicity in LDT_M_. To examine this hypothesis, we exposed LDT_M_ neurons to α-syn_M_ for 7 h in the presence of the GABA_A_, and GABA_B_ agonists, muscimol and baclofen (G-AGO), and cell death was compared to that in the other halves of the bisected slices exposed only to α-syn_M_. Remarkably, when exposed to α-syn_M_ the degree of cell survival seen in the halves of the slice treated with GABA receptor agonists was significantly higher than that in the other halves of the bisected slices exposed only to α-syn_M_ (Cell Survival: ⍺-syn_M_: 100.0 ± 2.3%, n_a_ = 168/n_40_ = 66, α-syn_M_ + G-AGO; 106.0 ± 2.7%, n_a_ = 127 /n_40_ = 48, p = 0.0334; Mann-Whitney Test; Fig. 5C). These results provide further support for the conclusion that in LDT_F_, a GABAergic-mediated mechanism protects against α-syn_M_-induced toxicity, and, excitingly, indicate that activation of GABA mechanisms could protect neurons of the LDT_M_ from α-syn_M_-mediated neurodegeneration.

## Discussion

We found that in contrast to what we previously saw in LDT_M_, effects of α-syn_M_ on the membrane of LDT_F_ neurons were inhibitory, and we recorded decreases in excitatory synaptic events, reductions in firing rate, and relatively more decreases in intracellular calcium. Further, cell death associated with α-syn_M_ was lower in females than in males. Changes in membrane currents and synaptic excitability noted in the SN did not exhibit a sex-based difference, suggesting nucleus specificity of α-syn_M_-mediated effects. Inhibitory membrane currents and reductions in calcium induced in LDT_F_ were found to involve inhibitory transmission, which when blocked revealed membrane excitation similar to that seen in LDT_M_. Finally, consistent with a protective role of inhibitory signaling, blockade of GABA_A_, GABA_B_, and glycine neurotransmission in the LDT of the female resulted in greater cell death and activation of GABAergic receptors reduced α-syn_M_-mediated neurodegeneration in the LDT of the male.

While the polarity of α-syn_M_-induced membrane effects on LDT_F_ neurons was opposite from that seen in our earlier study conducted in LDT_M_, when presynaptic input was blocked, an excitatory membrane response similar to that seen in LDT_M_ neurons was revealed. This leads us to the interpretation that α-syn_M_ induces a dual effect on the membrane of neurons of the LDT_F_ with the summation resulting in inhibition of membrane currents of the postsynaptic neuron. The mechanism underlying the occlusion of excitatory membrane actions putatively involved GABA-releasing, presynaptic neurons, although glycinergic mechanisms were not ruled out. Sex-based differences were also found in the polarity of calcium responses between male and female in that a decrease of intracellular calcium was observed in the majority of neurons of the LDT_F_, whereas in LDT_M_, the majority of responses were indicative of rises in intracellular calcium. Similar to the membrane responses, the decrease in intracellular calcium induced by α-syn_M_ in female involved inhibitory amino acids, suggesting sex-based differences in inhibitory transmission in the LDT. Cell death associated with α-syn_M_ was lower in females but increased when GABA was blocked suggesting that GABA is involved in inhibiting neurodegenerative processes. Consistent with this, the presence of GABA receptor acting agents was able to prevent neurodegeneration in the male LDT.

Besides suggesting potential targets to inhibit neurodegeneration, our data suggest presence of a GABAergic system in the female LDT, which leads to reductions in excitatory cellular effects and cell death and that this system does not function similarly in the male LDT. While not examined specifically within the LDT, the GABAergic system has shown sexual dimorphism in the brain. In both young and adult mice, expression of proteins involved in GABA synthesis and metabolism, as well as presence of GABA_A_ receptors have shown sex-based differences [30–35]. Further, the numbers of GABAergic neurons, as well as the responsiveness to GABA-acting drugs, have been shown to be associated with sex [36, 37]. The sex-specific phenotypic and functional differences in the GABAergic system may play key roles in the differential sensitivity of the LDT_M_ and LDT_F_ to α-syn_M_ [31, 34, 38, 39].

Our study has several limitations. Patch clamp recordings and multiple-cell calcium imaging with Fura-AM is difficult in slices from old animals, and thus it remains unknown if the sex-dependent effect of α-syn_M_ continues across ontogeny, which is relevant to neurodegeneration which is expected to increase across age. Further, we did not identify the phenotype of cells that were protected in presence of GABA_A_, and GABA_B_ receptor agonists. Since loss of cholinergic cells in the LDT and the neighboring pedunculopontine tegmentum has been one neuropathological feature noted in α-syn-related diseases [40, 41], we tried to target large neurons with the cholinergic profile [24], however, while we do not believe we recorded from many, if any, GABA cells as they are much smaller [17, 24], non-cholinergic neurons could have been included. Nevertheless, while we did not identify the LDT_F_ cell phenotypes exhibiting inhibitory membrane responses to α-syn_M_, we did show in our earlier work that excitatory cellular responses in the LDT_M_ were transmitter phenotype-independent [42]. Finally, while we did examine a nucleus which was central in SDs, given the global nature of sleep, it is almost certain that networks of nuclei, and not just activity in one nucleus mediate aberrant sleep behaviors seen in neurodegenerative diseases. Accordingly, future studies should examine effects across a larger age span, identify cellular phenotype, and conduct recordings across multiple sleep-controlling nuclei.

Despite the limitations, our work is based on multiple strengths, which differ from other investigations. Most studies of cellular effects of α-syn have been conducted using the oligomeric form of α-syn, and thus our data provides important information about effects of the monomeric form. Further, while the focus of much work has been on targeting α-syn intracellular exposure, we have utilized extracellular exposure. Additionally, in many studies, concentrations applied have been higher than those seen during pathological conditions (from 0.5 µM to 5 µM) [43]; however, we have used nanomolar concentrations of highly purified α-syn_M_, which more accurately reflects the clinical condition. Moreover, few studies have used ex vivo brain tissue but rather cultured cells; thus, our findings more directly add to the body of knowledge of effects in native mammalian tissue. Finally, to the best of our knowledge, no one has previously reported a sex-based difference in cellular effects of α-syn_M_ on any neuronal type in ex vivo studies. α-syn_M_ was shown to induce an inhibitory effect in synaptic transmission in Calyx of Held; however, while both male and female rats were used, data were not analyzed for potential sex-based differences [43]. Taken together, this constitutes the first report to show that α-syn_M_ at a concentration reflective of clinical exposures induces a sex-dependent, cellular effect in mammalian neurons. Furthermore, as no difference in membrane effects was observed between LDT_F_ and SN_F_, but we did see differences between the LDT_M_ and SN_M_ in earlier work [23], this suggests that α-syn_M_ induces sex-dependent effects in specific brain nuclei. Future studies of α-syn_M_ effects should consider sex as a factor as well as regional differences.

### Functional implications

The observed sex-based, different cellular responses likely have pervasive functional implications for behaviors and symptoms when neurons of the LDT are exposed to α-syn_M_. We have a working hypothesis that prodromal SDs in PD could be due to early dysfunction of sleep controlling nuclei, including the LDT, which has been implicated in RBD and EDS [23, 42, 44, 45].

Central to this hypothesis, we suspect that as levels of the monomeric form of α-syn rise, cellular effects are exerted on LDT neurons, which include enhancement of cellular excitability and increases in levels of intracellular calcium. Such effects are not elicited in the SN by the monomeric form, and it is believed to be the later appearing forms of aggregated oligomeric and fibril α-syn which produces neurodegeneration in the SN [23, 46–50]. Sustained elevation of excitability and calcium levels in the LDT induced by α-syn_M_ could trigger neurodegenerative processes when cells are excited for extended periods, and when calcium levels remain high [51, 52]. Consistent with this, we have shown that the effects induced by α-syn_M_ were associated with neuronal death in LDT_M_ neurons. However, in SN_M_ neurons in which α-syn_M_ induced an inhibitory membrane effect, and the predominant response was a decrease in intracellular calcium, no differences in neuronal survival were noted, which shows that α-syn_M_ cellular actions do not necessarily lead to degeneration as we saw in the LDT [23].

Interestingly, in the LDT_F_, α-syn_M_ induced very similar effects on the membrane and synaptic events to those seen in the SN_M_ leading us to suggest that these effects are neuroprotective in the female LDT. This tenant is supported by greater cell viability in the LDT of the female compared to that in the male following α-syn_M_ exposure. The neuroprotective mechanisms involved inhibitory amino acid transmission as the blockade of GABA and glycine receptors revealed an excitatory effect in the LDT_F_ similar to that seen in LDT_M_, which we hypothesize could underlie cellular degeneration. The outward current was sufficient to mask the concurrent excitatory effect and presumably limit putative damage from α-syn_M_-mediated excitation. Further, treatment with GABA receptor agonists resulted in reductions in cell death in the LDT_M_ lending further support to the interpretation that GABA signaling is neuroprotective. We did not identify the source of the putative, protective GABAergic effect in LDT_F_; however, elucidation of the source of this GABA tone in LDT_F_ is of great interest and will be a focus of future studies. Also likely contributing to α-syn_M_-induced neurodegeneration in the male LDT were the sex-dependent differences in the firing frequency, as a reduction in firing was induced in the LDT_F_ by α-syn_M_, whereas an enhancement in firing was seen in our earlier study in the LDT_M_ [42]. Over the long-term, increases of neuronal discharge can produce overexcitability-induced cell death since high-levels of excitability and firing elevates glutamate exposure, which results in alterations of intracellular calcium levels. This can trigger apoptotic events and collapse of mitochondrial functions which are all processes contributing to neuronal death [53–58]. Accordingly, the α-syn_M_-induced reduction in neuronal firing seen in LDT_F_ could exert a protective effect.

One implication of our findings is that processes controlled by the LDT that could be affected in PD are less likely to be affected in females. While speculative, our data do support clinical findings related to occurrence of LDT-involved sleeping disorders seen prodromal to PD. RBD and EDS appear to be more common in PD males as well as in males during the prodromal phase. The majority of patients diagnosed with RBD are male, with the reported percentage of females in these studies ranging from 10 to 17.5% of all diagnosed cases [9, 10, 45, 59–62]. Although very few studies have focused on differences in SD symptoms between men and women, sex differences in RBD symptomatology have been reported with aggressive and violent motor active RBD behaviors appearing more commonly in men than in women [63, 64]. EDS is characterized by the incapacity of the individual to stay awake during the circadian day due to excessive sleepiness. Several studies have examined the risk of development of PD in EDS patients; however, the majority of investigations which have shown an association between EDS in the prodromal phase of PD have been conducted in males [13, 65]. In one of the few studies to include both sexes, a higher risk of development of PD was documented in those expressing sleepiness during the day; however, the data were not analyzed to compare the risk in males vs. females, and the majority of the cohort who exhibited EDS were males, which reflects the sex-based odds ratio of PD in the general population [66]. In several studies of PD diagnosed patients, EDS has been shown to be more common among PD-affected men than women [65, 67, 68].

## Conclusion

Taken together, our data lead us to conclude that the cellular effects exerted by α-syn_M_ are neuroprotective in the LDT_F_ and could be sufficient to delay α-syn_M_-mediated cell death in this nucleus, perhaps ceasing when the relevant GABAergic neurons perish as neuronal loss proceeds throughout the PD affected brain. Output from the LDT to rostral and caudal targets is implicated in control of arousal during wakefulness, governance of the sleep and wakefulness cycle, and maintenance of motor atonia, which is a hallmark of REM sleep [18, 69]. Thus, if GABAergic neurotransmission plays a neuroprotective role by leading to outward currents and reductions in intracellular calcium, thereby counterbalancing negative effects induced by PD processes, this could block loss of cells in the LDT that produce motor atonia during REM sleep and an aroused EEG during wakefulness and sleep, and thereby lead to the lower frequency of RBD and EDS symptoms see in female when compared to those seen in male patients with PD [10, 70, 71].

As we observed no sex-based difference in cellular responses in the SN, our findings cannot account for the higher incidence of PD in males compared to females; however, we do suggest a mechanistic basis for the higher prevalence of SDs among male vs. female PD patients. Thus, our findings represent an important step toward the identification of sex differences in the mechanisms underlying the pathology of α-syn-associated neurogenerative diseases. Such identification offers the potential of targeting inhibitory mechanisms as neuroprotective strategies in neurodegenerative diseases, and to speed efforts for development of new directions for PD treatment and management in the prodromal phase of these diseases.

## Data Availability

All data are available upon reasonable request made to the corresponding author.
